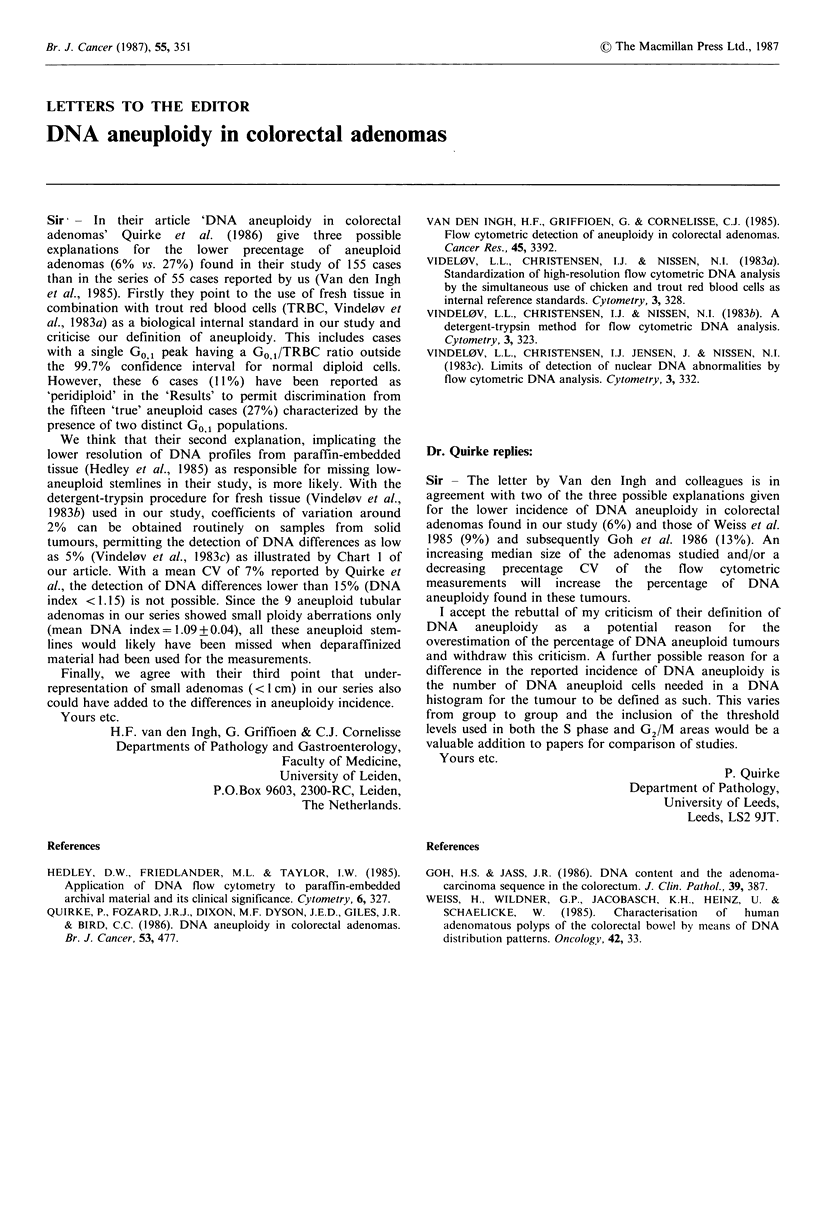# DNA aneuploidy in colorectal adenomas.

**DOI:** 10.1038/bjc.1987.66

**Published:** 1987-03

**Authors:** H. F. van den Ingh, G. Griffioen, C. J. Cornelisse


 © The Macmillan Press Ltd., 1987

### LETTERS TO THE EDITOR

### DNA aneuploidy in colorectal adenomas

**Sir** – In their article 'DNA aneuploidy in colorectal adenomas' Quirke *et al.* (1986) give three possible explanations for the lower precentage of aneuploid adenomas (6% *vs.* 27%) found in their study of 155 cases than in the series of 55 cases reported by us (Van den Ingh *et al.*, 1985). Firstly they point to the use of fresh tissue in combination with trout red blood cells (TRBC, Vindeløv *et al.*, 1983*a*) as a biological internal standard in our study and criticise our definition of aneuploidy. This includes cases with a single $G_{0,1}$ peak having a $G_{0,1}$/TRBC ratio outside the 99.7% confidence interval for normal diploid cells. However, these 6 cases (11%) have been reported as 'peridiploid' in the 'Results' to permit discrimination from the fifteen 'true' aneuploid cases (27%) characterized by the presence of two distinct $G_{0,1}$ populations.

We think that their second explanation, implicating the lower resolution of DNA profiles from paraffin-embedded tissue (Hedley *et al.*, 1985) as responsible for missing low-aneuploid stemlines in their study, is more likely. With the detergent-trypsin procedure for fresh tissue (Vindeløv *et al.*, 1983*b*) used in our study, coefficients of variation around 2% can be obtained routinely on samples from solid tumours, permitting the detection of DNA differences as low as 5% (Vindeløv *et al.*, 1983*c*) as illustrated by Chart 1 of our article. With a mean CV of 7% reported by Quirke *et al.*, the detection of DNA differences lower than 15% (DNA index $<1.15$) is not possible. Since the 9 aneuploid tubular adenomas in our series showed small ploidy aberrations only (mean DNA index $= 1.09 \pm 0.04$), all these aneuploid stemlines would likely have been missed when deparaffinized material had been used for the measurements.

Finally, we agree with their third point that under-representation of small adenomas ($<1$ cm) in our series also could have added to the differences in aneuploidy incidence.

Yours etc.

H.F. van den Ingh, G. Griffioen & C.J. Cornelisse
Departments of Pathology and Gastroenterology,
Faculty of Medicine,
University of Leiden,
P.O.Box 9603, 2300-RC, Leiden,
The Netherlands.

### References

HEDLEY, D.W., FRIEDLANDER, M.L. & TAYLOR, I.W. (1985). Application of DNA flow cytometry to paraffin-embedded archival material and its clinical significance. *Cytometry*, **6**, 327.

QUIRKE, P., FOZARD, J.R.J., DIXON, M.F. DYSON, J.E.D., GILES, J.R. & BIRD, C.C. (1986). DNA aneuploidy in colorectal adenomas. *Br. J. Cancer*, **53**, 477.

VAN DEN INGH, H.F., GRIFFIOEN, G. & CORNELISSE, C.J. (1985). Flow cytometric detection of aneuploidy in colorectal adenomas. *Cancer Res.*, **45**, 3392.

VIDELØV, L.L., CHRISTENSEN, I.J. & NISSEN, N.I. (1983*a*). Standardization of high-resolution flow cytometric DNA analysis by the simultaneous use of chicken and trout red blood cells as internal reference standards. *Cytometry*, **3**, 328.

VINDELØV, L.L., CHRISTENSEN, I.J. & NISSEN, N.I. (1983*b*). A detergent-trypsin method for flow cytometric DNA analysis. *Cytometry*, **3**, 323.

VINDELØV, L.L., CHRISTENSEN, I.J. JENSEN, J. & NISSEN, N.I. (1983*c*). Limits of detection of nuclear DNA abnormalities by flow cytometric DNA analysis. *Cytometry*, **3**, 332.